# Tuning the Physicochemical, Structural, and Antimicrobial Attributes of Whey-Based Poly (L-Lactic Acid) (PLLA) Films by Chitosan Nanoparticles

**DOI:** 10.3389/fnut.2022.880520

**Published:** 2022-04-28

**Authors:** Farhad Garavand, Milad Rouhi, Shima Jafarzadeh, Diako Khodaei, Ilaria Cacciotti, Masoumeh Zargar, Seyed Hadi Razavi

**Affiliations:** ^1^Department of Food Chemistry and Technology, Teagasc Moorepark Food Research Centre, County Cork, Ireland; ^2^Bioprocess Engineering Laboratory (BPEL), Department of Food Science, Engineering and Technology, College of Agriculture and Natural Resources, University of Tehran, Karaj, Iran; ^3^Department of Food Science and Technology, School of Nutrition Sciences and Food Technology, Research Center for Environmental Determinants of Health (RCEDH), Health Institute, Kermanshah University of Medical Sciences, Kermanshah, Iran; ^4^School of Engineering, Edith Cowan University, Joondalup, WA, Australia; ^5^Department of Sport, Exercise, and Nutrition, Galway-Mayo Institute of Technology (GMIT), Galway, Ireland; ^6^Department of Engineering, INSTM RU, University of Rome “Niccolò Cusano”, Rome, Italy

**Keywords:** poly-l-lactic acid, fermentation, whey, chitosan nanoparticle, food packaging

## Abstract

Recently, the research and innovation to produce raw materials from microbial processes has gained much attention due to their economic and environmental impacts. Lactic acid is a very important microbial product due to its wide application in the food, pharmaceutical, cosmetic, and chemical industries. In the current study, poly (L-lactic acid) (PLLA) was produced by the ring opening polymerization (ROP) technique of L-lactic acid recovered from whey fermentation, and was used for the production of nanocomposites films reinforced with chitosan nanoparticles (CNPs) (average diameter ca. 100–200 nm). Three different CNPs concentrations, namely 1, 3, and 5% w/w, were tested, and their influence on the physical, mechanical, thermal, antibacterial and structural attributes of PLLA film was assessed. The results showed that the addition of CNPs up to 3% caused a significant improvement in water vapor permeability, appearance, tensile strength and elongation at break. The antibacterial properties of nanocomposites followed a dose-depended pattern as a result of CNPs addition. Therefore, the best inhibitory effects on *Escherichia coli* and *Staphylococcus aureus* was made by the addition of 5% of CNPs and lower dosages slightly affected the growth of pathogens or didn't cause any inhibitory effects (in 1% of CNPs). It can be concluded that the incorporation of CNPs into the PLLA matrix allows to improve the structural, thermal, physical, mechanical and antibacterial properties of the polymer, generating promising systems for food packaging and biomedical applications.

## Introduction

Films and coatings for food packaging applications are aimed at protecting the food products during the storage by retarding the migration of moisture, carbon dioxide, and oxygen. Prohibiting the migration of odors, colorants, physical protection, handling, and improving shelf life are some of the other requirements for ideal food packaging (1). These films can be also used as carriers for antimicrobial, antioxidants, vitamins, probiotics, colorants, and nutrients (2). Petroleum based polymers are commonly used for packaging, even if they are generally non-biodegradable and their disposal in landfills and oceans causes serious environmental issues (3). Hence, the research and innovation for developing bio-based polymers to reduce the use of petroleum-based polymers has gained much attention (4). Many researches have been carried out for producing biodegradable packaging materials, in terms of films and coatings, based on biopolymers (starch, whey, proteins, chitosan, cellulose, poly (lactic acid) (PLA)) (5). The films and coatings used in food packaging should be completely biodegradable in industrial or home composting conditions and degrade into water, carbon dioxide, methane, and organic materials by the selected microorganisms which PLA-based films possess this advantage while their biodegradation rate is still < the biopolymer films such as gelatin and sodium caseinate (6).

PLA, an aliphatic polyester from lactic acid or lactide monomers, is one of the most studied and prevalent biodegradable polymers with potential applications in several sectors (7), mainly for packaging materials, fibers, and scaffolds (8). PLA is manufactured by the controlled fermentation of carbohydrates from different bio-sources such as corn starch or sugarcane. PLA films have good mechanical and barrier properties but the high production cost for PLA compared to the petroleum based polymers is limiting its wide application in different industries (9). On the other hand, producing PLA is accompanied by a high amount of water footprint (0.248 m^3^/kg) that is mainly associated with maize cultivation. Utilization of edible and nutritive plants such as corn, cassava, sugarcane, and sugar beet pulp is another drawback attributed to the industrial production of PLA. Therefore, production of PLA by the fermentation of food by-products such as whey is a suggested to optimize the industrial production of this biopolymer (10).

Indeed, lactic acid can be generated from chemical production or from fermentation pathways by lactic acid bacteria (LAB). The chemical synthesis generally leads to a racemic mixture of DL-lactic acids (10), whereas the fermentation process presents many advantages with respect to the chemical one: the capability of using renewable and cheap substrates, lower production temperature, lower energy consumption, as well as the possibility to produce pure L or D lactic acid forms. The extraction and purification of organic acids are generally difficult and time consuming and include extraction with chemical solvents followed by purification through filtration, centrifugation, crystallization, and chromatography (11).

Due to the high cost of equipment and the use of high volume of toxic solvents in the mentioned methods, the separation of organic compounds, such as amino acids, organic acids, some vitamins, dyes, etc., from the biological sources using emulsion liquid membrane technique (ELM) has attracted a lot of attention in the recent years (12). Indeed, the isolation of organic compounds by ELM technique has many advantages, including high solvent transfer flux, high specificity, minimal use of toxic organic solvents and their recyclability, and the replacement of conventional organic solvents (13). The liquid membrane system consists of a diluent, a surfactant to stabilize the emulsion, and a carrier to separate the solutes by chemical reaction. In the internal aqueous phase, the presence of a stripping agent is necessary to keep the desired compound inside the liquid membrane (14). Thus, soluble substances are able to diffuse through the membrane phase and move from the outer phase to the inner phase (either due to their solubility or by reacting with carrier molecules and forming a solute-carrier complex). Extraction of L-lactic acid from fermentation broth by ELM systems has been successfully carried out with high purity (15).

Moreover, to improve the mechanical properties, thermal stability, and barrier properties of bio-nanocomposite films, a possible strategy is the incorporation of nanoparticles into the biopolymer matrix. Some nanoparticles can also act as antimicrobial agents and extend the shelf-life of food products by inhibition of microbial growth. These nanoparticles can be composed of natural biopolymers such as chitosan, cellulose, chitin, or of metals such as silver or zinc (3, 5, 16). Chitosan nanoparticles (CNPs) are widely studied in food industry as antimicrobial agents due to their good antimicrobial properties, while they can improve some structural, mechanical, and physical attributes of packaging films (17).

**Scheme 1:**
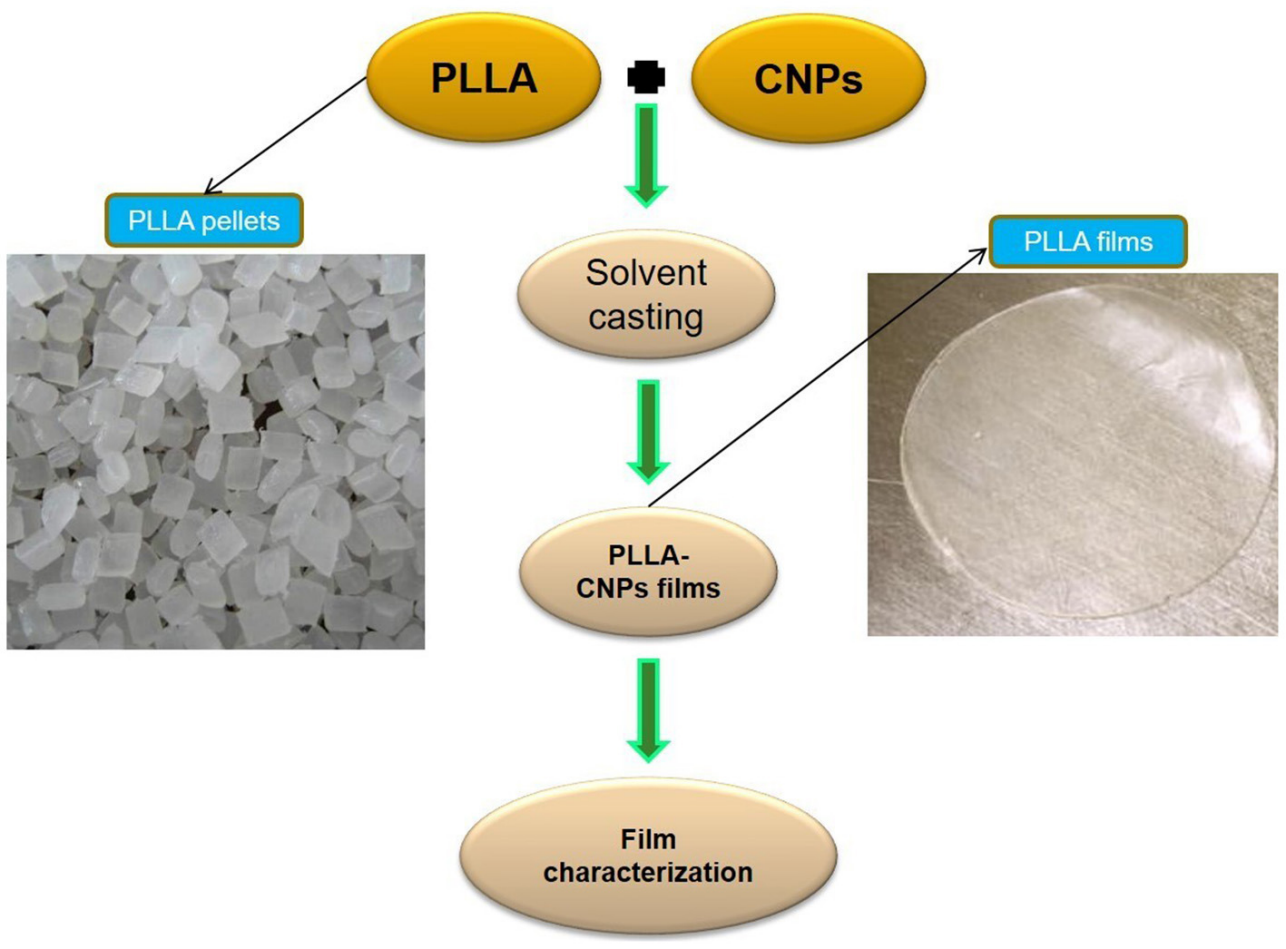
Schematic drawing of the production of PLLA-CNPs nanocomposites.

The main aim of this research was to study the characteristics of PLLA prepared from the polymerization of L-lactide monomers produced by whey fermentation, and evaluate the influence of CNPs incorporation with different concentrations (i.e., 1, 3, and 5% w/w) on physical, mechanical, barrier, and antimicrobial properties on PLLA films (Scheme 1), in comparison with commercial PLA.

## Materials and Methods

### Materials

Whey powder and sunflower oil were obtained from Borna Laban Dairy (Tehran, Iran), and Nina Co. (Tehran, Iran), respectively. Natureworks Ingeo-4043D PLA (Minnetonka, MN, USA) with a density of 1.2 g.cm^−3^ was used as control sample in this study. Dichloromethane (DCM), chitosan (medium molecular weight, with deacetylation degree of 75–85% and molecular weight of 190–310 kDa), acetic acid, tripolyphosphate (TPP), sorbitan monooleate (Span 80), Aliquat 336, yeast extract, MRS agar, and tin octanoate (SnOct_2_) were purchased from Sigma-Aldrich (Steinheim, Germany). *Lactobacillus paracasei, Lactobacillus casei, Lactobacillus delbrueckii, Escherichia coli* (*E. coli*), and *Staphylococcus aureus* (*S. aureus*) were obtained from Persian Type Culture Collection (PTCC) (Tehran, Iran). Other chemicals and reagents were of analytical grade.

### Methods

#### Microbial Fermentation

Whey microbial fermentation was performed according to our previous research (15). Briefly, a whey solution (6% w/v in distilled water) was heated at 90°C for 20 min using a heater stirrer (IKA, Germany). The heated solution was cooled down to 25°C and centrifuged at 4,000 × *g* for 15 min and then the supernatant was collected as fermentation broth. The collected supernatant (2 L) was supplemented by yeast extract (5 g/L), potassium hydrogen phosphate (3 g/L), di-potassium hydrogen phosphate (3 g/L), manganese (II) sulfate monohydrate (0.3 g/L), and magnesium sulfate (0.5 g/L). The supplemented supernatant was first autoclaved at 121°C for 15 min, and then inoculated with *Lactobacillus paracasei, Lactobacillus casei*, and *Lactobacillus delbrueckii*. To enumerate the bacteria population three samples were taken (each 3 mL), 1 mL of each sample was decimally diluted in sterile Ringers' solution, and then plated on MRS agar at 37°C for 48 h according to Charalampopoulos et al. (18) with slight changes. Colonies were then counted and expressed as log_10_ colony-forming units (CFU)/mL.

#### Recovery of Lactic Acid Using Emulsion Liquid Membrane

An emulsion liquid membrane system was engaged to extract lactic acid from fermentation broth as described by Garavand et al. (15). Span 80, NaOH, Aliquat 336, and sunflower oil were used as the surfactant, entrapment agent, carrier (extractor), and liquid membrane, respectively. Firstly, NaOH was added to double distilled water and the membrane was prepared by the gradually addition of sunflower oil, Span 80, and Aliquat 336. The alkalized aqueous solution was considered as the internal aqueous phase and the mixture of surfactant, oil, and carrier was regarded as the liquid membrane phase at the 1:1 internal aqueous phase to liquid membrane phase ratio. In order to prepare the liquid membrane, an Ultra-Turrax dispersing machine (IKA, Germany) was applied to mix surfactant, oil, and carrier thoroughly, while the internal aqueous phase was gradually added for 5 min until a stable emulsion was obtained. The prepared emulsion was exposed to fermented whey and gently mixed using a propeller to enhance the extraction efficiency. The following operational parameters were used to extract lactic acid from fermented whey as described by Garavand et al. (15): exposure time of 10 min, propeller speed of 300 rpm, and ELM fabricated by using 4% w/w Span 80, 3% w/w carrier, and NaOH 0.1 N. After the end of the exposure time, the process stopped and the emulsion was separated from the fermented broth as per their phase differences, and finally the produced emulsion (ELM extractor) was de-emulsified by using freezing method to break the membrane and internal aqueous phases enriched with lactic acid.

#### Determination of L-Lactic Acid Concentration

The L-lactic acid concentration was determined using high-performance liquid chromatography (HPLC) equipped with CHIROBIOTIC column (15 cm length, 2.1 mm inner diameter, and the particles size of 5 μm). The mobile phase was prepared from 15% (v/v) ammonium acetate (33.3 mmol in water) and 85% (v/v) of acetonitrile. The selected solutions were degassed and passed through 0.45 μm filters and then injected into the column with a speed of 0.5 mm/min using a pump. The flow rate of mobile phase was set at 0.7 mL/min.

#### Polymerization of the L-Lactic Acid Monomers

The extracted L-lactic acid by ELM technique with over 90% purity was utilized for the PLLA polymerization. Polymerization of L-lactic acid monomers was conducted using ring-opening polymerization (ROP) approach as described by Lopes and Jardini (19) with slight modifications. Firstly, L-lactic acid was added to a reaction flask equipped with a vacuum pump (set at 100 mbar), dehydrated at 100°C for 2 h, and then heated again at 160°C for 2 h to generate oligomers. In the next step, the process temperature was increased up to 210°C for 12 h, the obtained lactides were collected and dried at 40°C for 2 h under vacuum. The dried lactides were mixed with 1% w/w SnOct_2_ as catalyst at 140°C for 4 h under continuous stirring to induce the polymerization and obtain the PLLA polymer. The produced PLLA was dissolved in DCM and precipitated by methanol (98%), filtrated and dried. The molecular weight of the synthesized PLLA was characterized by using Brookhaven molecular weight analyser (BI-MwA) (Brookhaven Instruments, Holtsville, NY, USA) with multi-angle static light scattering detector, and tetrahydrofuran was used as mobile phase at flow rate of 1 mL/min. The functional groups and thermal properties of the obtained polymers were also determined by FTIR, and differential scanning calorimetry (DSC), respectively which are explained in details in the following sections.

#### Chitosan Nanoparticles Production

The CNPs were prepared based on ionotropic gelation between chitosan and TPP according to Vahedikia et al. (16). For this aim, 1 g of the chitosan powder was dissolved in 100 mL acetic acid solution (1% w/v in distilled water) into a beaker covered with aluminum foils and kept at 30°C for 24 h under continuous magnetic stirring at 400 rpm. The resulted solution was passed through Whatman No. 3 and then filtered by 0.45 μm microfilters. Afterwards, 1% (w/v) solution of TPA was gradually added over 40 min to the chitosan-TPP solution at 1,000 rpm at the room temperature. The prepared suspension was then centrifuged at 1,000 × *g* for 20 min at 4°C, the supernatant was discarded and the CNPs pellets were collected, washed twice with distilled water and then freeze dried for further analysis. The CNPs mean particle size and polydispersity index (PDI) were recorded using Zetasizer (Malvern Instruments, UK), based on dynamic light scattering technique, at 25°C. The CNPs morphology was examined by transmission electron microscopy (TEM) (JEOLJEM 200CX, Japan) at acceleration voltage of 30 kV. CNPs were stained with phosphotungstic acid onto a carbon-coated copper meshwork and oven-dried at 30°C for 2 h.

#### Composite Film Preparation

Since there is no previous reference on this neat PLLA, the solvent casting method on PLA film synthesis by Abdulkhani et al. (20) was used to fabricate commercial PLA (PLA-C), neat PLLA and PLLA-CNPs nanocomposite films. Briefly, 5 g PLLA (or PLA) were dissolved in 100 mL DCM under agitation at ambient temperature for 8 h. The prepared solution was then poured into Teflon dishes (10 cm diameter) and left under fume-hood at ambient temperature for 24 h. For loading CNPs into PLLA composites, PLLA solutions were prepared according to the already mentioned film making process (for 8 h) and then 1, 3, and 5% w/w (dry basis) CNPs were added to the solution and vigorously homogenized by using an Ultra-Turrax homogenizer for 15 min at 10,000 rpm, followed by sonication at ambient temperature for 30 min. The obtained suspensions were poured into Teflon dishes (10 cm diameter), and left under fume-hood at ambient temperature for 24 h. The prepared composite films were finally peeled off and kept in zip kips for further analyses and designed as PLLA-x%, where x is the CNPs concentration.

#### Characterization of the Produced Films

##### Physical Properties of the Films

The thickness of the films was measured using a digital micrometer (Mitutoyo, Tokyo, Japan) with an accuracy of 0.001 mm. For each sample, 10 points were measured to provide an average value. The water vapor permeability (WVP) was obtained following ASTM E96-00 standard test (21). Briefly, the fabricated films were cut into 2 cm diameter discs, surrounded on top of the vial cells by parafilm, and finally transferred to a desiccator saturated with K_2_SO_4_ to reach a constant high relative humidity (RH) of 97.3% at room temperature. The WVP (g.mm.kPa^−1^.h^−1^.m^−2^) of the produced films was calculated based on the weight gain of the permeation cells over time, using the following equation:


(1) $$\begin{array}{l} WVP=\frac{WVTR}{P\left(R_{1\;}-R_{2\;}\right)}\times X \end{array}$$

where WVTR is the water vapor transmission rate, calculated as the slope of the permeation (g/s) divided by the transfer zone (m^2^), X is the film thickness, P is the water vapor pressure at saturation state (Pa), and R_1_ and R_2_ are RH inside of desiccator and permeation cell, respectively.

The barrier attributes of the prepared films against visible and ultraviolet (UV) lights were determined at wavelength range of 200–800 nm according to Hosseini et al. (22). To do that, film samples were cut into 1 cm × 4 cm pieces, placed inside the cuvette and their absorbance was measured by using a UV/Vis spectrophotometer (Shimidzu, Kyoto, Japan). An empty cuvette was considered as control and the measurements performed in triplicate. The transparency of films was also calculated as follow:


(2) $$\begin{array}{l} Transparency\;\left(\frac{A}{mm}\right)=-\text{log}T/X \end{array}$$

where A is the absorbance at the specified wavelength, T is the transmittance (%) at the specified wavelength, and X is the film thickness (mm). According to the equation, a higher transparency index indicates higher film opacity.

The opacity of the films was also determined by the following formula:


(3) $$\begin{array}{l} Opacity=\text{Abs}600/X \end{array}$$

where Abs600 is the absorbance at 600 nm and X is the film thickness (mm).

Different color parameters (lightness (L^*^), red-green (a^*^) and yellow-blue (b^*^)) were also obtained using a Hunter lab colorimeter (Minolta CR 300 Series, Osaka, Japan) (23).

##### Mechanical Characterization of Films

The mechanical parameters of packaging composite films were measured based on Lizundia et al. (24). Briefly, composite films were cut into 1 *cm* × 10 cm pieces and set inside a desiccator enfolding with NaBr solution to maintain a RH of 57% for 3 days before the test. After measuring their moisture content, a texture analyzer apparatus (SMT5, Santam, Tehran, Iran) fitted out with 100 N load cell, 10 cm distance between grips, and the crosshead speed of 10 mm/min was employed to measure the mechanical properties.

##### X-Ray Diffraction

The XRD analysis was carried out by using a Philips PANalytical X'Pert diffractometer (Amsterdam, the Netherlands) with a CuKα radiation source. The scans were plotted in a 2θ diffraction angle oscillating mode ranging from 5° to 60° at the scan speed of 2°/min with a wavelength of 0.154 nm.

##### Film Microstructure

The surface morphology of composite films was observed by using a scanning electron microscopy (SEM) (Hitachi, Japan) at the accelerating voltage of 30 kV. The prepared films were coated with a gold layer by using an ion-sputter coater at room temperature prior to visualization.

##### Fourier Transform Infrared

A FTIR (Nicolet iS10, Thermo Scientific, Courtaboeuf, France) apparatus equipped with an attenuated total reflectance (ATR) cell was employed to detect the functional chemical groups of produced pellets and composite films. The pellets and films spectra were recorded in the wavenumber range of 4,000–400 cm^−1^, spectral resolution of 40 cm^−1^, and taking the average of 32 scans.

##### Thermal Properties

The thermal behavior of the fabricated pellets and films was examined by using a differential scanning calorimetry (DSC) (Q2000, TA Instrument, USA) with sample weight of 10 mg, temperature range of −50°C to +200°C, heating-cooling rate of 10°C/min, and nitrogen flux of 50 cc/min. The melting temperature (T_m_), glass transition temperature (T_g_), cold crystallization temperature (T_cc_), melting enthalpy (ΔH_m_), cold crystallization enthalpy (ΔH_cc_), and degree of crystallinity (χ) were obtained from both the first and the second heating scans. The χ value was calculated as follows:


(4) $$\begin{array}{l} \chi =\frac{\Delta H_{m}-\Delta H_{cc}}{\Delta H_{m}^{\infty }\left(1-x\right)} \end{array}$$

where $\Delta H_{m}^{\infty }$ is the melting enthalpy associated to 100% PLA (93 J/g) (9), and x is the CNPs weight fraction.

##### Antimicrobial Properties

The agar diffusion method was used to study the antibacterial impacts of the fabricated films against food-borne microorganisms (*E. coli* and *S. aureus*) (25). At first, the composite films were cut into 15 mm rings and then positioned on Brain Heart Infusion (BHI) agar plates. The plates were inoculated with 100 μL of the broth cultures of *E. coli* and *S. aureus* and then incubated at 37°C for 24 h in oven. Finally, the inhibition zone was measured by using a digital micrometer and the inhibition zone area was calculated.

### Statistical Analysis

The SPSS (V.18.1) software was employed to analyse the obtained data. The significant differences were determined by one-way analysis of variance (ANOVA) using Duncan multiple range test at the probability level of 5%. The experiments were performed in triplicate.

## Results and Discussions

### Lactic Acid Production

**Figure 1:**
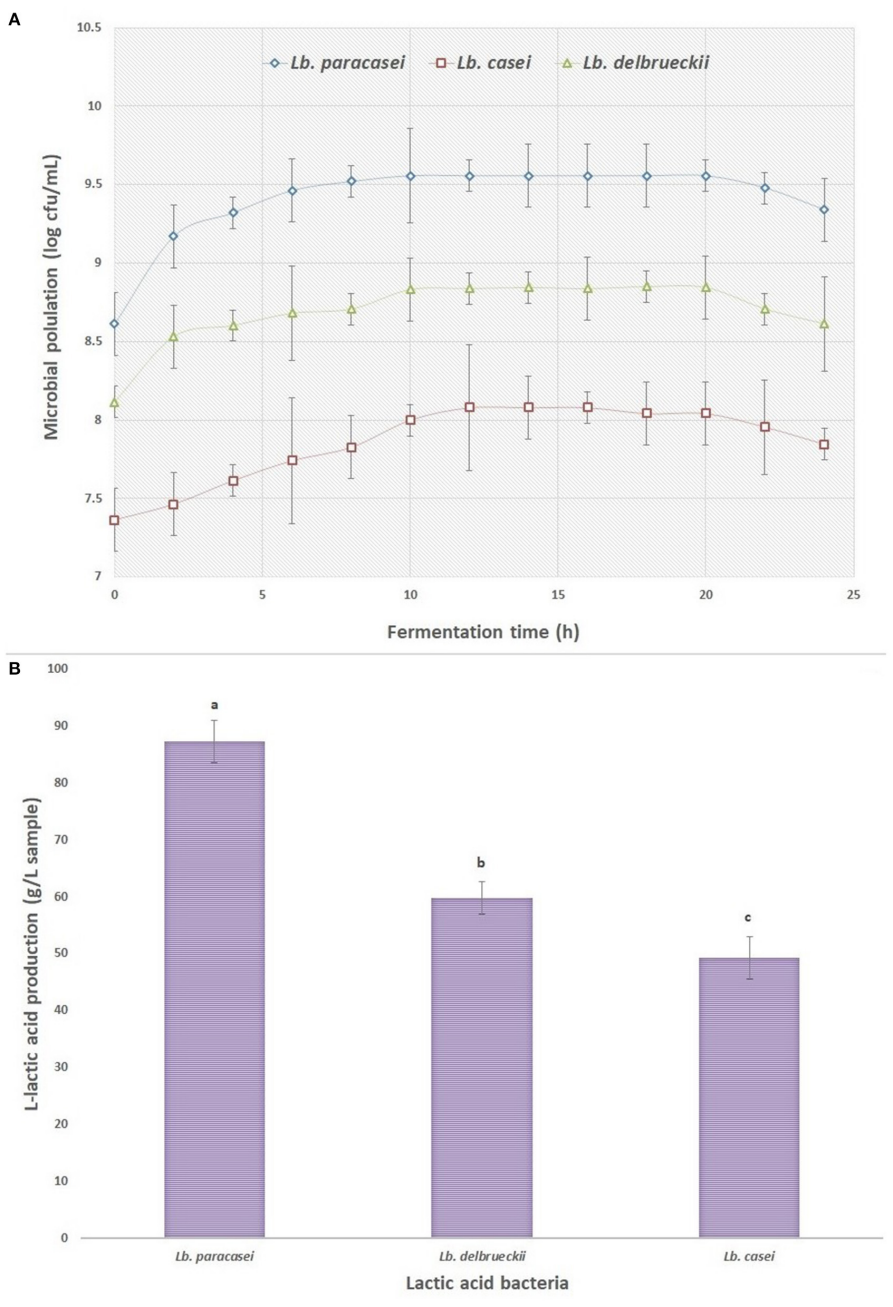
**(A)** The growth curve of different lactic acid bacteria inoculated to supplemented whey (three samples were taken at each time point), and **(B)** the amount of L-lactic acid produced by the lactic acid bacteria at the end of fermentation process.

Figure 1A shows the population of LAB inoculated into whey media during 24 h fermentation. As it can be seen, the population of the studied inocula was in log phase after 10–12 h fermentation, the LABs were entered into the stationary phase and the number of viable bacteria was declined after 24 h of fermentation. This latter experimental evidence could be attributed to the reaching bacteria to death stage and increasing acids in media, followed by reducing carbon and nitrogen sources. The low pH of the medium (ranging between 4.3 and 4.4) can result in lower growth rate and increase in latent growth phase of microorganisms (26). The amount of L-lactic acid produced by the three LABs was recorded after the end of the fermentation process and the results are shown in Figure 1B. As it is shown, the amount of L-lactic acid produced by the *Lb. paracasei* was significantly higher (87 g/L) than the other LAB strains, in correlation with the observations from growth rate curves (Figure 1A). Therefore, the fermented products from *Lb. paracasei* were selected for extraction stage via ELM system and the extracted pure L-lactic acid was used to synthesize PLLA by ROP method. Similar observations were reported by other researches in batch fermentation of different LABs. Panesar et al. (27) reported that the amount of L- lactic acid produced from the fermentation of whey by *Lb. casei* was 33.7 g/L. John et al. (28) stated that *Lb. delbrueckii* generated about 82 g/L of lactic acid from the batch fermentation of cassava bagasse.

### Characterization of the Synthesized PLLA

**Figure 2:**
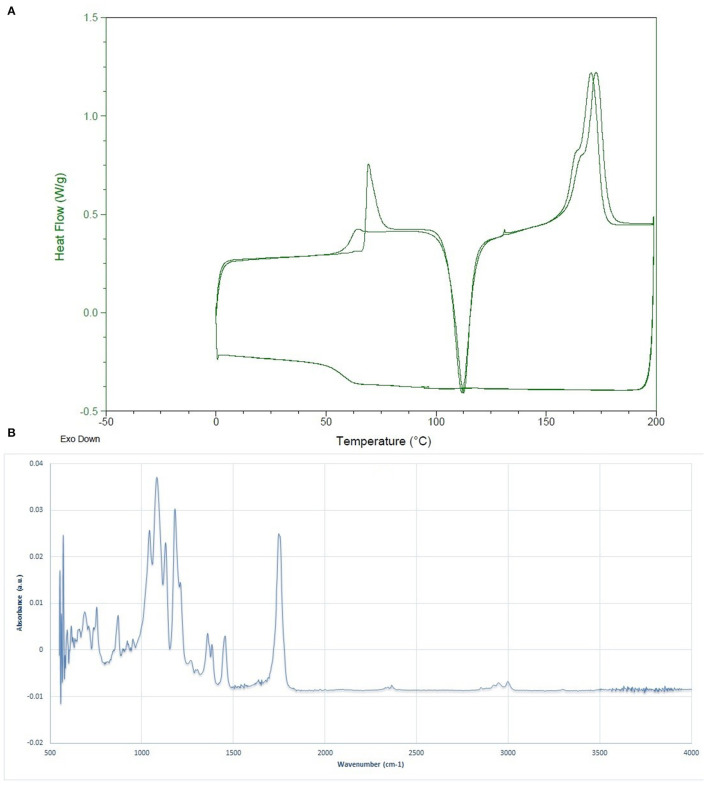
**(A)** DSC curve, and **(B)** FTIR spectrum of the synthesized poly (L-lactic acid) (PLLA).

The molecular weight determination results via BI-M_wA_ software showed that the Mw of the fabricated PLLA was 1,53,000 ± 2,400 Da, which is appropriate for food packaging applications. The thermal features of the synthesized whey-based PLLA are shown in Figure 2A. The thermal characterization of the produced PLLA was compared to commercial PLA, PDLLA, and PLLA, on the basis of data reported in scientific researchers in Table 1. As it can be seen, the polymers prepared in this study presented higher T_g_, T_c_, and T_m_ values compared to the commercial PLA, poly (L-, D-lactic acid) (PDLLA), and were comparable to PLLA. The FTIR spectrum of the PLLA synthesized in this study is shown in Figure 2B. The peaks observed at 2,998 and 2,951 cm^−1^ were attributed to the –CH_3_ symmetric and asymmetric vibrational stretchings, respectively. The bond at 1,784 cm^−1^ is also attributed to the C = O stretching and the peaks at 1,455 and 1,360 cm^−1^ were related to the –CH_3_ symmetric and asymmetric stretchings, respectively (31). The peaks at 1,181 and 1,131 cm^−1^ were ascribed to the C-O vibrational stretching. The results observed for PLLA in this study are in correlation with the results reported by Cheng et al. (32), Qin et al. (33) and Mofokeng and Luynt (29).

**Table 1:** Thermal properties of the poly (L-lactic acid) (PLLA) synthesized in this study compared to commercial PLA, PDLLA, and PLLA.

**Polymer**	**Glass transition temperature (**°**C)**	**Melting temperature (**°**C)**	**References**
PLLA	68	176.3	The current study
PLA-C	64	155.1	The current study
PLA	64	154	(29)
PLA	60	151	(30)
PDLLA	55	149	Polysciences, Inc.
PLLA	72	180–195	Biodegmer, Inc.
PLLA	60–65	173–178	Polysciences, Inc.

### Characterization of Chitosan Nanoparticles

**Figure 3:**
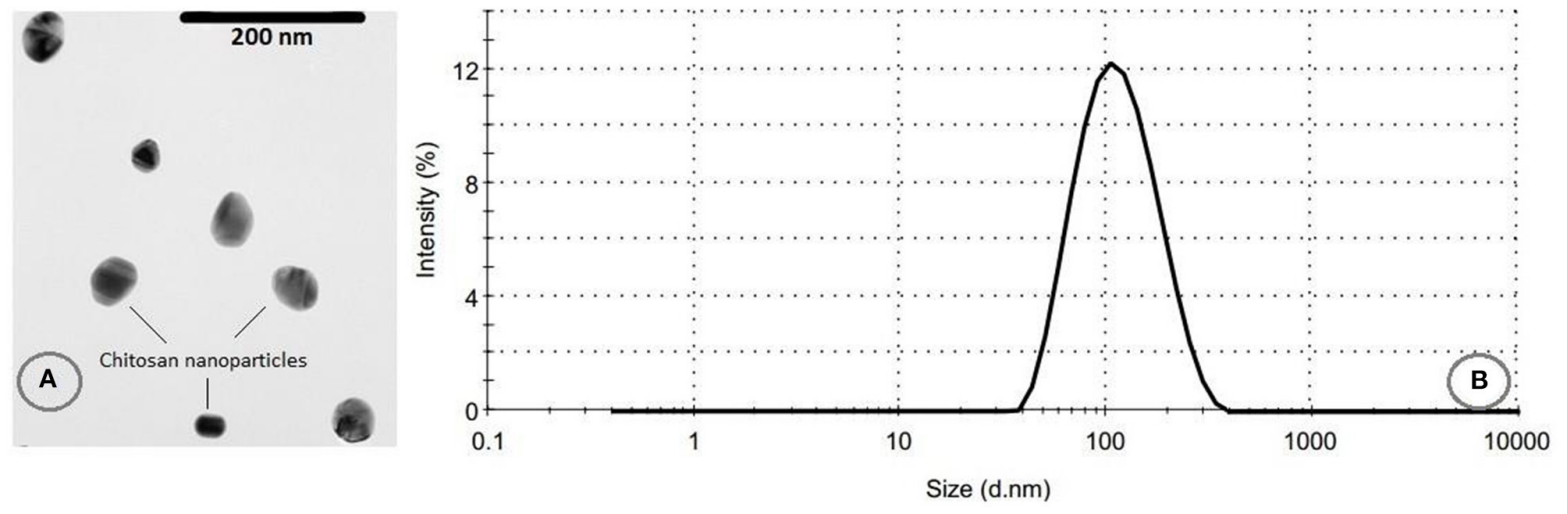
**(A)** TEM micrograph of freeze-dried CNPs, and **(B)** their particles size distribution.

The SEM micrographs of freeze-dried CNPs are presented in Figure 3A, revealing a size range of 50–70 nm, in good agreement with the observations by Antoniou et al. (17) and Chang et al. (34). The particles size distribution and the average particle sizes are also shown in Figure 3B. As it can be seen, the PDI was 0.235 and the average diameter of CNPs was 102.0 ± 5.9 nm. The low particle size distribution of the CNPs confirms the monodisperse size of the fabricated particles (35). The size of CNPs obtained by both methods were almost similar, even though the dried samples exhibited a smaller particle size compared to the hydrated CNPs. The possible explanation for this behavior is the swelling of CNPs in aqueous solution or aggregation of CNPs (22). Shapi'i et al. (36) reported that the particle size of CNPs produced via ionic gelation were in the range of 60–110 nm which are similar to the results from the current study.

### Characterization of CNPs- Loaded PLLA Films

#### Physical Properties

Table 2 shows the physical properties of the films including the thickness, WVP, and color characteristics. The thickness plays a significant effect on the physical and mechanical properties of films, especially on the barrier properties against the moisture and gases (37). From Table 2, the thickness of the control films (i.e., PLA-C and PLLA) and CNPs loaded films was in the range 70–80 μm and no significant differences were revealed (*p* > 0.05), in agreement with Abdulkhani et al. (20) who reported a thickness value of 75 μm for PLA films. It can be concluded that the CNPs were homogeneously distributed within the polymer matrix and filled the voids inside the polymer chains, so no significant thickness change was observed between PLA, PLLA and nanocomposite films, even though a slight increment of the thickness with the addition of CNPs, in terms of average value, was detected, as expected.

**Table 2:** Physical properties of poly (L-lactic acid) (PLLA) nanocomposite films, compared to neat PLA and PLLA films.

**Film samples**	**PLA-C**	**PLLA**	**PLLA-1% CNPs**	**PLLA-3% CNPs**	**PLLA-5% CNPs**
Thickness (μm)	70.98 ± 4.07^a^	74.23 ± 5.06^a^	80.55 ± 9.39^a^	77.57 ± 5.34^a^	79.66 ± 6.67^a^
L*	68.35 ± 2.26^a^	55.07 ± 1.25^b^	51.14 ± 4.01^c^	46.08 ± 3.09^d^	40.22 ± 2.54^e^
a*	−2.07 ± 0.26^a^	−1.66 ± 0.29^a^	−0.40 ± 0.05^b^	+0.14 ± 0.03^c^	+0.49 ± 0.07^d^
b*	−3.11 ± 0.17^d^	−3.19 ± 0.09^d^	−3.77 ± 0.15^c^	−4.29 ± 0.19^b^	−5.07 ± 0.14^a^
VWP (10^−11^gm/m^2^sPa)	1.93 ± 0.06^a^	1.88 ± 0.05^a^	1.29 ± 0.11^b^	1.20 ± 0.07^b^	1.01 ± 0.09^c^

*Experiments were performed in triplicate for three film samples in each treatment*.

*Values are presented as mean ± std. deviation*.

*Values with the same letter in each column are not significantly different (p > 0.05)*.

The color of the films has a significant influence on the acceptance of packaging material by the consumers. The color differences between biopolymers are mainly attributed to the biopolymer kind, pH, presence of additives (plasticizers, nanoparticles, etc.), heating process conditions, method used for preparation of films, etc. The color characterization of the films showed that by incorporation of CNPs, the lightness of the films (L^*^) reduced, as expected. The L^*^ in control PLA and PLLA films were 68 and 55, respectively, and it was significantly reduced in the presence of CNPs. The lowest L^*^ value was observed in PLLA film contained 5% CNPs. The a^*^ values of films were increased by the addition of higher percentages of CNPs into the films, while b^*^ values decreased. Similarly, Antoniou et al. (17) reported that the CNPs increased the a^*^ value in tara gum films, due to the nature of chitosan extracted from crab's skin that has a yellowish color. It should be noted that these films were almost colorless as the a^*^ and b^*^ values were close to 0.

The moisture has a direct impact on the chemical reactions and microbial contamination of food products. Therefore, the WVTR and WVP are important factors for the packaging films (38). The WVP and WVTR of the different films are presented in Table 2. As evident, the control PLA and PLLA films exhibited the highest (1.93 × 10^−11^ gm/m^2^sPa) and lowest (1.88 × 10^−11^ gm/m^2^sPa) WVP, respectively, in correlation with the observations reported by Huang et al. (39) for pure PLA films containing 98% of L- lactide isomers (1.89 × 10^−11^ gm/m^2^sPa). Moreover, the incorporation of CNPs (1–5% w/w) to the PLLA films significantly reduced the WVP from 1.29 × 10^−11^ to 1.01 × 10^−11^ gm/m^2^sPa. It can be suggested that the CNPs addition increased the compactness of the films structure and reduced the transportation of moisture and gases from the surface of the film, by filling the voids within polymer's matrix (40, 41). The permeation of moisture from biopolymer films and the rate of transfer are dependent to the nature of biopolymers, hydrophobicity, the presence of different ingredients in film's matrix, voids, cracks and the integrity of the polymer's structure (42). It seems that loading CNPs into PLLA films were not able to change their characteristic from low barrier to medium or high barrier films as all mentioned values, in another words, the PLLA films continue being considered as lower water vapor barrier films when loaded with CNPs.

Similarly to the color, the transparency and optic barrier properties of the films also exert a direct effect on the acceptance of the food material by the consumers. The most influential factors on the transparency of the films are the nature of the selected biopolymers and the functional groups in the polymers chains (43). The transparency values of the films at different wavelengths are collected in Table 3. The UV-transparency for the films (at 200–280 nm) was relatively low and no significant difference between PLA and PLLA was observed. However, by incorporation of the CNPs within the polymeric matrix, a significant decrease in light transparency at 280 nm occurred and the lowest value was detected for the films loaded with the highest amount of CNPs (a 10% transparency decrease in PLLA films containing 5% w/w of CNPs compared to the control film). Due to the insolubility of CNPs in the PLA, these nanoparticles are probably able to fill the voids in the polymer matrix, leading to a lower light transparency. Indeed, the opacity of the nanocomposite films might reflect the UV light and reduce the transparency of the films. Therefore, it can be concluded that the CNPs loaded PLLA films prohibited the transmittance of the UV light which could induce the lipid oxidation in the food systems (44). Similarly, the nanocomposite films showed a lower light transparency to the visible lights (350–800 nm, Table 3) compared to the control films. The CNPs aggregation and filling the voids in polymer matrix are the possible reasons for blocking the visible light, which is in good agreement with Hosseini et al. (22).

**Table 3:** Optical properties of poly (L-lactic acid) (PLLA) nanocomposite films, compared to neat PLA and PLLA films.

**Film samples**	**Light transmission at different wavelengths (%)**							**Transparency**
	**200**	**280**	**350**	**500**	**600**	**700**	**800**	
PLA-C	0	23.0 ± 1.2^a^	55.7 ± 1.1^a^	68.3 ± 2.1^a^	74.7 ± 2.0^a^	77.3 ± 2.2^a^	81.7 ± 4.0^a^	1.07 ± 0.11^d^
PLLA	0	22.4 ± 0.7^a^	50.6 ± 1.5^b^	65.0 ± 3.7^a^	73.6 ± 3.5^a^	75.0 ± 2.8^a^	87.4 ± 2.7^a^	1.13 ± 0.18^d^
PLLA-1% CNPs	0	17.1 ± 0.6^b^	43.2 ± 1.7^c^	59.1 ± 2.1^b^	63.2 ± 2.6^b^	67.1 ± 3.8^b^	71.2 ± 2.1^b^	1.63 ± 0.21^c^
PLLA-3% CNPs	0	14.9 ± 0.5^c^	40.9 ± 1.5^c^	55.8 ± 3.1^bc^	56.1 ± 3.5^c^	60.2 ± 2.4^c^	66.1 ± 2.5^c^	2.55 ± 0.15^b^
PLLA-5% CNPs	0	12.3 ± 0.9^d^	36.8 ± 1.9^d^	50.0 ± 4.2^c^	52.8 ± 4.9^c^	55.5 ± 1.3^d^	60.8 ± 2.7^d^	3.34 ± 0.14^a^

*Values are presented as mean ± std. deviation*.

*Values with the same letter in each column are not significantly different (p > 0.05)*.

#### Morphological Studies

**Figure 4:**
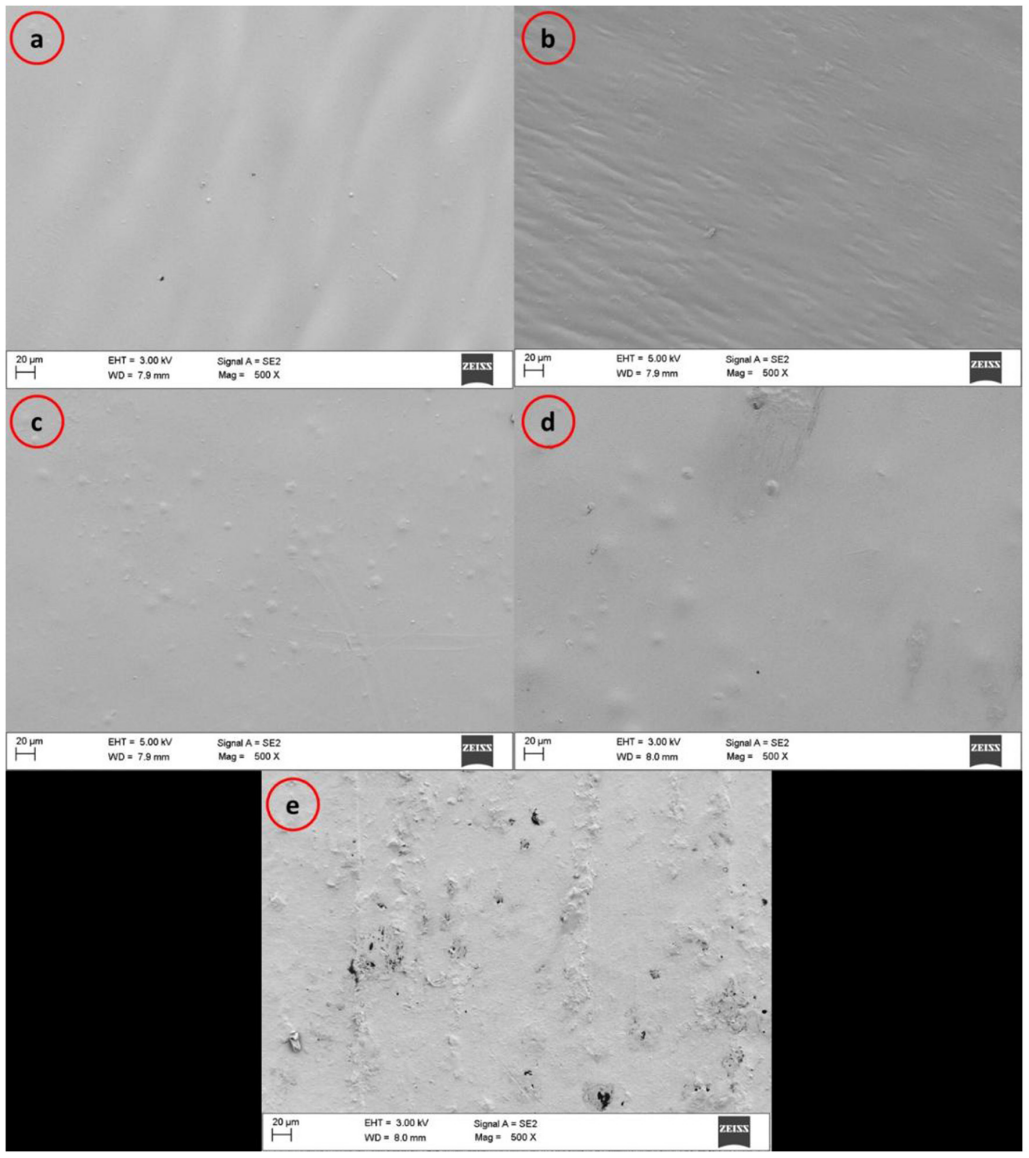
SEM micrographs of surfaces of **(a)** commercial PLA films (PLA-C), **(b)** PLA films manufactured from fermentation process (poly(L-lactic acid) (PLLA)), **(c)** PLLA-1% CNPs, **(d)** PLLA-3% CNPs, **(e)** PLLA-5% CNPs.

The permeability of packaging polymers to moisture and gases can be influenced by the polymer matrix morphology and its homogeneity (37). Figure 4 shows the SEM micrographs of the nanocomposite films surface. The commercial PLA (PLA-C), PLLA prepared from fermentation process (PLLA), and the films containing 1 and 3% of CNPs exhibited a smooth surface without any considerable roughness. Antoniou et al. (17) also reported that the uniform dispersion of CNPs in tara gum films led to a homogeneous structure of the polymer matrix and to their improved physical, thermal, and mechanical properties, due to the CNPs filling of voids between the polymer chains. Hosseini et al. (45) observed that CNPs incorporation in low amounts (2% w/w) allowed a uniform dispersion within fish gelatin matrix without aggregations, while at the higher amounts of 6 w/w and 8% w/w, medium interconnections and aggregations in some regions were observed, respectively. Chang et al. (34) reported a similar observation for starch films loaded with different amounts of CNPs. Shapi'i et al. (36) observed a very good CNPs dispersion within starch matrix at different concentrations (0–20% w/w) with no agglomeration. On the other hand, the film containing 5% w/w of CNPs showed a rough surface and loss of homogeneity, due to the CNPs aggregation in some regions of the film, as evident in Figure 4e.

#### XRD Pattern

**Figure 5:**
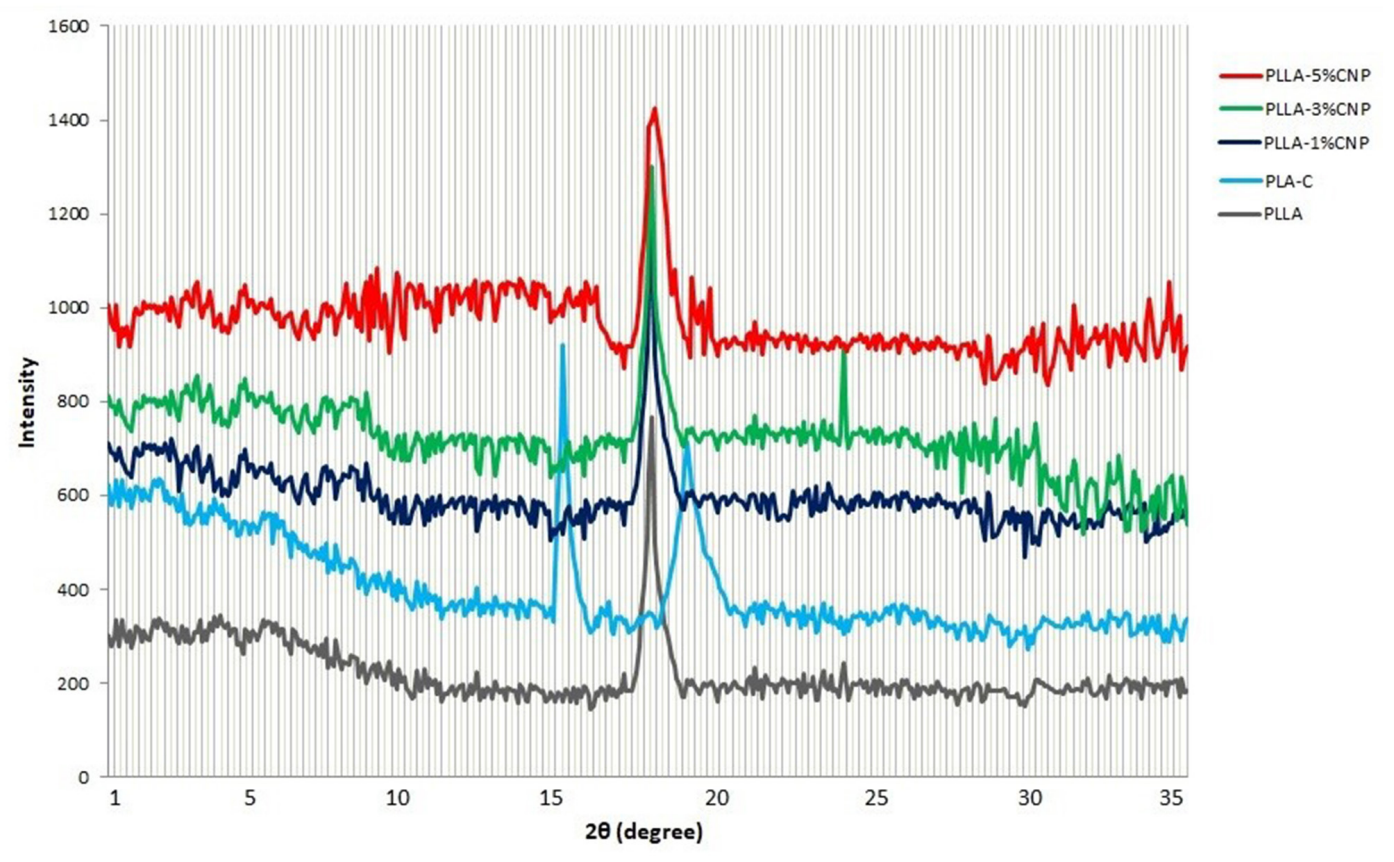
X-ray diffraction patterns of commercial PLA films (PLA-C), PLA films manufactured from fermentation process (poly(L-lactic acid) (PLLA)), PLLA-1% CNPs, PLLA-3% CNPs, PLLA-5% CNPs.

Figure 5 shows the XRD patterns of PLA-C, PLLA and PLLA nanocomposite films. Interestingly, PLA-C and fermentation derived PLLA showed two different patterns. The film manufactured from PLA-C showed sharp and wide peaks at 14.7° and 18.1°, respectively, whereas the films made from synthesized PLLA only presented a sharp peak at 17.2°. In the case of CNPs loaded PLLA films, similar peaks related to neat PLLA were observed. However, the intensity and the length of the peaks increased with the addition of CNPs. Nanocomposite containing 3% w/w of CNPs exhibited a weak peak at 32° and the addition of higher amount of CNPs (5% w/w) did not significantly influence the length of the peak. Indeed, chitosan powders present an intense sharp peak at the range of 18–20° which is generally due to the high degree of chitosan crystallinity (46). Cui et al. (47) reported the molecular interactions between CNPs and zein protein which exhibited a weak broad peak at 2θ of 20°. The presence of CNPs also increased the crystallinity of the zein films containing cinnamon essential oils (16). Pantani et al. (48) studied the XRD patterns of PLA films loaded with zinc nanoparticles (Zn-NPs) and they observed a peak at 17° which was attributed to the orthorhombic crystals. They also reported that by increasing Zn-NPs amount in PLA films, especially at the concentrations of 2–3% w/v, peaks at 31.6° and 6.2° appeared, confirming the improvement in the polymer structure. Dadashi et al. (49) reported that the incorporation of nanoclays into PLA polymer increased the distance between polymer chains and improved the crystallinity of the biopolymer. However, microcrystalline cellulose nanoparticles (MC-NPs) had no effect on the XRD pattern of the PLA films (up to 7% w/w incorporation), very probably due to the destruction of MC-NPs crystalline structure after addition to the PLA (50). These authors reported that the peaks for PLA film at 12.6°, 22.4°, 22.5°, 38.6°, and 39.5° are mainly attributed to the semi-crystalline structure of PLA. However, the number and the position of these peaks are different than commercial and also PLLA polymers in this study.

By incorporation of CNPs to the film matrix, the intensity of peaks (especially for 1 and 3% w/w) increased and also the peaks shifted to the higher degrees which can be due to the presence of CNPs with high crystallinity in film matrix. These results can be also attributed to the reactions between CNPs and PLA chains which affect the structure of matrix. It can be concluded that the film contained 5% w/w of CNPs has aggregations in some regions that lead to lower structural order.

#### FTIR Analysis

**Figure 6:**
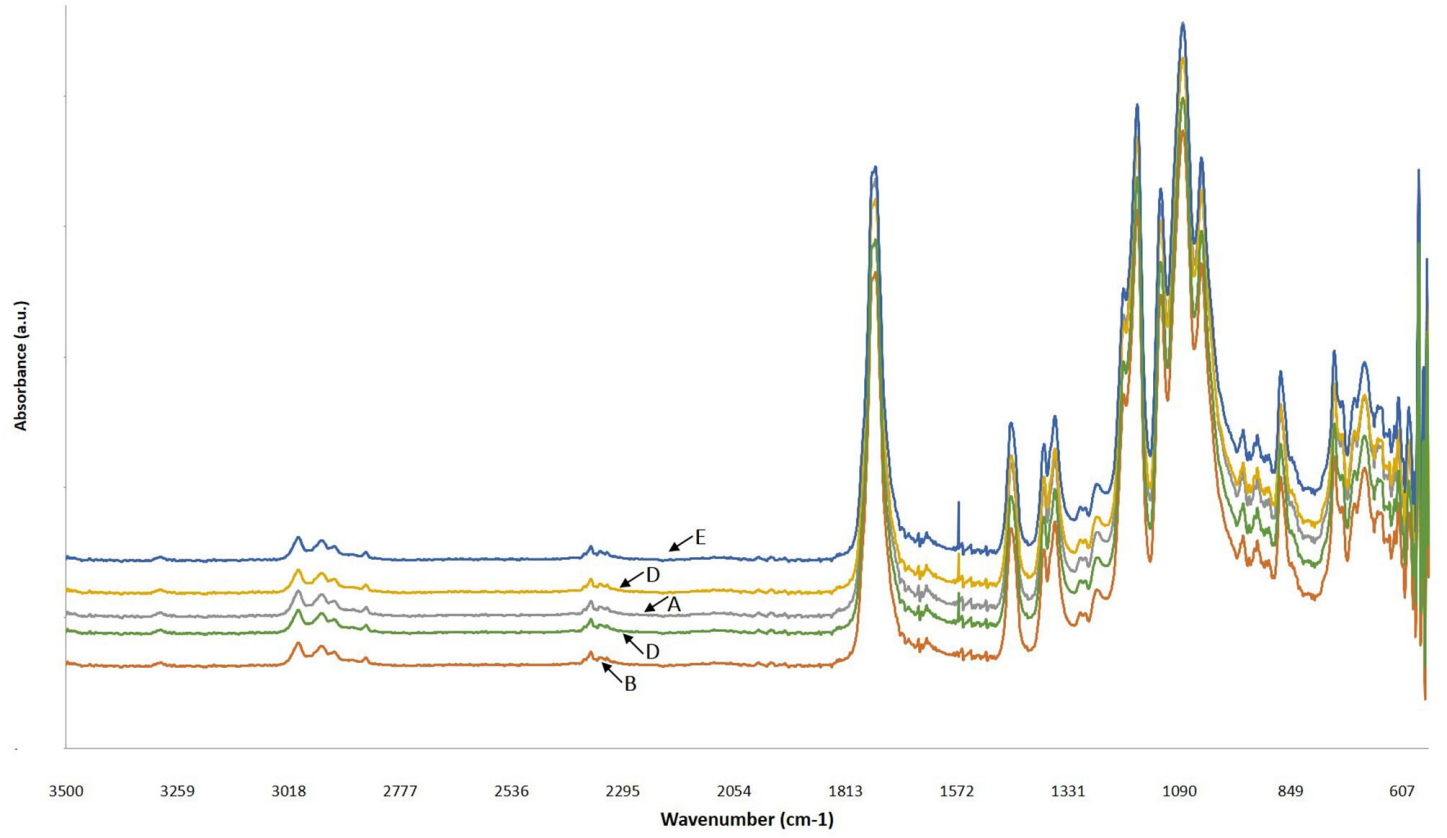
FTIR spectra of (A) commercial PLA films (PLA-C), (B) PLA films manufactured from fermentation process (poly(L-lactic acid) (PLLA)), (C) PLLA-1% CNPs, (D) PLLA-3% CNPs, (E) PLLA-5% CNPs.

As it can be seen in Figure 6, the FTIR spectra of all PLA and PLLA-based films, controls and composites, were similar and they all showed weak peaks at 2,998 and 2,951 cm^−1^ which are the vibrational frequency for symmetric and asymmetric –CH_3_ stretching, respectively. The peak observed at 1,748 cm^−1^ was attributed to the stretching in C=O bond and peaks at 1,455 and 1,360 cm^−1^ were related to symmetric and asymmetric stretching of –CH_3_, respectively (51). The frequencies observed at 1,181 and 1,131 cm^−1^ were ascribed to the vibrational stretching of C-O and the peak at 870 cm^−1^ was related to the C-C vibrational stretching (52). These observations are in agreement with the reports by other researches about different PLA films (29, 31). The bond observed at the wavelength of 1,567 cm^−1^ in the spectra of the PLLA/CNPs films was attributed to the vibrational stretching of amine groups of the CNPs (17, 53) and shifted to higher positions by increasing the CNPs amount. This could be due to the bonding between CNPs and the carboxyl side-chains of the PLLA. Similarly, Antonino et al. reported that the addition of CNPs to tara gum films shifted the vibrational stretching at wavelength of 1,559 to 1,567 cm^−1^ and that the related peak intensity enhanced increasing the amount of CNPs (17). Therefore, the bond at 1,559 cm^−1^, related to the amide bonds, was replaced with the new peak at 1,576 cm^−1^ that it is attributed to the interactions between PLA and CNPs.

#### Thermal Analysis

The results from DSC analysis of PLLA and nanocomposite films containing 1–5% w/w of CNPs are shown in Table 4. As it can be seen, a significant difference between the thermal properties of the studied films was observed. The T_g_ of PLA-C film was 64°C, significantly lower than the T_g_ of PLLA (68°C), and the addition of CNPs led to a slight decrease in PLLA glass transition temperature. The T_m_ of the films showed a similar trend with T_g_ so that a slight decrease in T_m_ was observed in the films by increasing CNPs amount. T_m_ of the PLLA films was significantly (+15°C) higher than the PLA-C. The addition of the CNPs probably speeds up the crystallization process of PLLA due to the NPs nucleating properties, and therefore reduces the melting temperature of nanocomposites. Frone et al. (54) reported that cellulose nanoparticles cause a reduction in the melting temperature of PLA films by acting as nucleating agents. Similarly, the reduction in T_m_ of fish gelatin nanocomposite films reinforced with CNPs (2–8% w/w) was reported by Hosseini et al. (45).

**Table 4:** Thermal properties of poly (L-lactic acid) (PLLA) nanocomposite films, compared to neat PLA and PLLA films.

**Film samples**	**Cold crystallization**				**Melting**		**Melting crystallization**		***X* (%)**	**Tg (**°**C)**
	**T_**cc1**_ (**°**C)**	**ΔH_**cc1**_ (J/g)**	**T_**cc2**_ (**°**C)**	**ΔH_**cc2**_ (J/g)**	**T_**m**_ (**°**C)**	**ΔH_**m**_ (J/g)**	**T_**c**_ (**°**C)**	**ΔH_**c**_ (J/g)**		
PLA–C	100.5	−37.5	150.4	−4.1	155.1	12.6	99.3	−12.5	26.9	64.0
PLLA	115.1	−34.4	169.1	−3.8	176.3	29.8	111.3	−29.9	64.1	68.0
PLLA-1% CNPs	114.7	−31.9	167.8	−3.3	174.5	29.2	110.6	−29.3	62.9	67.6
PLLA-3% CNPs	114.0	−30.2	170.3	−2.7	172.9	29.0	108.2	−29.2	62.5	67.2
PLLA-5% CNPs	112.2	−29.8	167.9	−2.5	169.9	25.5	109.5	−26.2	52.7	66.9

Moreover, PLA-C and neat PLLA DSC thermograms presented two melting peaks while the first peak in nanocomposites was weaker and shifted to lower temperatures. The presence of two melting peaks in neat PLA can be attributed to the melting behavior of polymer followed by re-crystallization (10). Moreover, the weak peaks in nanocomposite films can be related to the presence of CNPs which altered the PLA crystal structure. Cui et al. (47) reported that the CNPs enhanced the thermal stability of zein nanocomposite films.

As presented in Table 4, the melting enthalpy of neat PLA-C and PLLA were 12.6 and 29.8 J/g, respectively, and CNPs addition in 1 and 3% w/w didn't change the enthalpy of PLLA films, while the addition of 5% w/w of CNPs reduced the melting enthalpy to 25.5 J/g. The crystallization enthalpies were similar to the melting enthalpies and it can be concluded that the thermal history of PLA films will be faded after first cycle of heating and cooling in DSC curve, taking into account that the sum between ΔH_m_ and ΔH_c_ was ≈ 0 (55). Concerning the calculated percentage of crystallization degree, the addition of lower CNPs concentrations (1 and 3% w/w) did not led to significant difference, while by the addition of 5% w/w a significant decrease was observed in crystallinity of PLLA films. Farah et al. (56) reported that the PLA containing more than 90% of PLLA has more tendencies to crystalize, whereas the tendency to the amorphous form increases decreasing the PLLA content. Therefore, it can be suggested that probably the impurities, such as different fillers (plasticizers, nanoparticles, etc.), or the racemic lactic acid can reduce the crystallization of the PLA. Another study showed that addition of 5% of cellulose nanoparticles to the PLA film caused a reduction in crystallization from 39 to 34% (57).

#### Mechanical Properties

The mechanical parameters of the films are presented in Table 5. No significant differences were detected between the commercial PLA films and PLLA films prepared in this study, in terms of tensile strength (TS), Young modulus (YM) and elongation at break (EAB). The addition of CNPs increased the TS of the films, as well as the YM, and decreased the EAB, as expected. In details, the highest TS and YM values were achieved for the films containing 3% w/w of CNPs with the tensile strength of 50.2 MPa vs. 38.7 MPa for the control films, and the Young modulus of 2.28 GPa vs. 1.40 GPa for the control films. However, no further significant increase in TS and YM of the composite films was observed by addition of higher amounts of CNPs (i.e. 5% w/w). These observations are in consistent with our previous work where the incorporation of CNPs (2–4%w/w) and cinnamon essential oil (CEO) into the zein films improved the tensile strength of the composite films, with a consequent decrease of the composite films EAB (16). Similarly, Chang et al. (34) reported that the CNPs dispersion within the starch polymer containing glycerol improved the film TS.

**Table 5:** Mechanical properties of poly (L-lactic acid) (PLLA) nanocomposite films, compared to neat PLA and PLLA films.

**Film samples**	**Tensile strength (MPa)**	**Young modulus (GPa)**	**Elongation at break (%)**
PLA-C	40.8 ± 1.9^bc^	1.43 ± 0.12^b^	30.7 ± 2.0^c^
PLLA	38.7 ± 2.0^c^	1.40 ± 0.17^b^	25.6 ± 3.5^c^
PLLA-1% CNPs	44.3 ± 3.0^b^	2.11 ± 0.16^a^	50.2 ± 1.6^b^
PLLA-3% CNPs	50.2 ± 2.3^a^	2.28 ± 0.10^a^	60.1 ± 3.5^a^
PLLA-5% CNPs	43.1 ± 1.7^bc^	2.01 ± 0.23^a^	18.8 ± 2.9^d^

*Values are presented as mean ± std. deviation*.

*Values with the same letter in each column are not significantly different (p > 0.05)*.

Moreover, the EAB of the films increased by the addition of CNPs in the range 1–3% w/w. Similar observation was reported by De Moura et al. (40) who confirmed that the elongation modulus of the hydroxypropyl methylcellulose (HPMC) films was increased by the addition of CNPs. On the other hand, the sample loaded with 5% w/w of CNPs showed a lower EAB value. Thus, the CNPs dispersion inside the polymer matrix and filling the void spaces can cause a reduction in elasticity of films (47, 58). The reduced EAB at concentration of 5% w/w of CNPs could be ascribed to the voids reduction within the polymeric chains and to the increase in intramolecular interactions (45, 59).

It can be suggested that the CNPs distribution in lower amounts was more homogenous, as expected, leading to more compatibility with hydrophobic PLLA. The incorporation of higher amounts of CNPs reduced the integrity of polymer's matrix and reduced the mechanical properties of the films, as confirmed by the films microstructure analysis (Figure 4). Therefore, the faults in mechanical properties of 5% w/w CNPs loaded PLA films can be explained by the non-homogenous structure of these films. Moreover, the reduction in some physical and mechanical properties of 5% CNPs loaded PLLA films contained could be ascribed to its lower crystallinity compared to 1 and 3% CNPs loaded samples (23), as testified by the lower crystallinity degree calculated from DSC data (Table 4).

#### Antimicrobial Properties

Table 6 shows the antimicrobial properties of PLA-C, PLLA, and nanocomposite PLLA/CNPs films. As it was predicted, the PLA-C and PLLA films showed no antibacterial activities against *E. coli* and *S. aureus*. It seems that the addition of 1% w/w of CNPs to the PLLA films had no effect on the growth of selected bacteria, while the higher amounts of CNPs (3 and 5% w/w) significantly reduced the number of viable bacteria in the growth media, achieving the highest inhibitory effect for PLLA film loaded with 5% w/w of CNPs. The results showed that the CNPs had more inhibitory effect on the gram negative bacteria (*E. coli*) compared to the gram positive (*S. aureus*) one. de Boer et al. (60) reported that the PLA films loaded with 1–3% of chitin-NPs had no inhibitory effect on food-borne bacteria. The antimicrobial activity at the doses of 3 and 5% w/w is probably due to the electrostatic interactions between the protonated ${\text{NH}}_{3}^{+}$ groups of CNPs and the electronegative charges on the surface of bacteria that lead into releasing protein compounds and other cell parts of bacteria that have lethal effect on the bacteria (61). Abdou et al. (62) reported that the addition of CNPs in 2–4% w/w had considerable antimicrobial properties against *S. aureus* and coliform bacteria. Shapi'i et al. (36) also reported that starch-based nanocomposite films containing CNPs exhibited inhibitory activity against *B. cereus, S. aureus*, and *E. coli*, while the control starch films showed no inhibitory activity against the selected microorganisms. They stated that the increase in CNPs concentration from 0 to 20% w/w in starch films led to enhanced inhibition zone area under the films for gram-positive bacteria.

**Table 6:** Antimicrobial properties of poly (L-lactic acid) (PLLA) nanocomposite films, compared to neat PLA and PLLA films.

**Film samples**	**Inhibitory zone after 18 h (mm)**	
	***E. coli***	***S. aureus***
PLA-C	ND	ND
PLLA	ND	ND
PLLA-1% CNPs	ND	ND
PLLA-3% CNPs	3.2 ± 0.3^b^	2.7 ± 0.1^b^
PLLA-5% CNPs	5.1 ± 0.4^a^	4.0 ± 0.3^a^

*Values are presented as mean ± std. deviation. ND, not detected*.

*Values with the same letter in each column are not significantly different (p > 0.05)*.

## Conclusions

In this study PLLA and CNPs (1–5% w/w) based nanocomposite films were successfully prepared. SEM micrographs demonstrated that the PLLA films containing 1 and 3% of CNPs exhibited a smooth surface without any considerable roughness. Incorporation of CNPs up to 3% w/w allowed to improve the water vapor permeability of the films, tensile strength, flexibility, and elastic modulus of the films. On the basis of the collected results, the CNPs addition can improve the functional properties of biodegradable films for food and medical applications. However, the lower flexibility compared to the neat PLA films, and mainly to the conventional polymers, such as polyvinylchloride (PVC) or polyethylene (PE), might limit their industrial application. Therefore, more attempts are necessary to increase the functional properties of these polymers for further industrial applications.

## Data Availability Statement

The original contributions presented in the study are included in the article/supplementary material, further inquiries can be directed to the corresponding authors.

## Author Contributions

FG: conceptualization, methodology, and roles/writing—original draft. MR: data curation and investigation. SJ: formal analysis, resources, and software. DK: visualization and roles/writing—original draft. MZ: writing—review and editing. IC: funding acquisition, investigation, supervision, and writing—review and editing. SR: conceptualization, project administration, and supervision. All authors contributed to the article and approved the submitted version.

## Conflict of Interest

The authors declare that the research was conducted in the absence of any commercial or financial relationships that could be construed as a potential conflict of interest.

## Publisher's Note

All claims expressed in this article are solely those of the authors and do not necessarily represent those of their affiliated organizations, or those of the publisher, the editors and the reviewers. Any product that may be evaluated in this article, or claim that may be made by its manufacturer, is not guaranteed or endorsed by the publisher.
